# The natural menstrual cycle revisited – can natural cycle be trusted

**DOI:** 10.1186/s13048-024-01469-2

**Published:** 2024-07-22

**Authors:** B Alsbjerg, US Kesmodel, P Humaidan, L Bungum

**Affiliations:** 1 The Fertility Clinic, Skive Regional Hospital, Resenvej 25, Skive, Denmark; 2 Department of Clinical Medicine, Aarhus University, Aarhus, Denmark; 3 Department of Obstetrics and Gynaecology, Aalborg University Hospital, Aalborg, Denmark; 4 Department of Clinical Medicine, Aalborg University, Aalborg, Denmark; 5 Reproductive medicine, Lund University, Malmö, Sweden

**Keywords:** Natural cycle, Progesterone, Estradiol, Luteal phase, Regular menstrual

## Abstract

**Background:**

The serum progesterone (P4) level during the luteal phase (LP) plays a crucial role in the initiation and maintenance of pregnancy. However, it is unclear whether the natural cycle consistently provides the best endocrine profile and whether mid-luteal serum P4 levels are always sufficient to support implantation and early pregnancy. The question has become more relevant in relation to fertility treatment, as more frozen embryo transfer cycles are performed in the natural cycle. Moreover, can serum hormone levels and covariates measured during the follicular phase (FP), such as Follicle Stimulation Hormone (FSH), Luteinizing Hormone (LH), Estradiol (E2), Anti-Mullerian Hormone (AMH) and Antral Follicle Count (AFC), be used to predict P4 levels during the luteal phase (LP)?

**Results:**

This observational prospective cohort study analysed 26 healthy women with a cycle length between 21–35 days and a body mass index (BMI) < 30 kg/m^2^. Blood sampling started on the fifth day of the menstrual cycle and continued every fifth day until the next cycle. The procedure was repeated for a total of three cycles. The study found that only ten women had a P4 level greater than 30 nmol/L on cycle day 20 or 25 in all three cycles. In total, only 45 cycles out of 77 cycles had serum P4 levels ≥ 30 nmol/L.

The E2 level ≥ 345 pmol/L on cycle day 10 proved to be predictive of a P4 level of ≥ 30 nmol/L on either day 20 or day 25 with a sensitivity of 57% and a specificity of 89%. No other covariates, including the FSH level cycle day 5, LH levels during the follicular phase, age, weight, AFC and AMH cycle day 5 correlated with LP P4 levels.

**Conclusions:**

A significant correlation between FP E2 levels cycle day 5 (> 131pmol/L) and cycle day 10 (> 345pmol/L) and a LP P4 level ≥ 30 nmol/l was found; thus, the FP E2 level is a predictor of corpus luteum competence. Our findings highlight the existence of suboptimal P4 levels during the LP and a significant inter-individual and intra-cycle variation in P4 levels during the LP in regular menstruating women.

**Supplementary Information:**

The online version contains supplementary material available at 10.1186/s13048-024-01469-2.

## Introduction

Over the past decade there has been a significant increase in the number of frozen embryo transfer cycles, with natural cycle frozen embryo transfer becoming more prevalent than hormone replacement therapy frozen embryo transfer (HRT-FET), due to a lower incidence of hypertensive disorders and pre-eclampsia compared to HRT [25]. Nevertheless, it remains unclear whether the natural cycle consistently offers the best endocrine profile and whether mid-luteal serum progesterone (P4) levels are always sufficient to support implantation and early pregnancy.

The P4 level during the luteal phase (LP) plays a crucial role in the initiation and maintenance of pregnancy. Its primary source is the corpus luteum (CL), which constitutes the remnants of the postovulatory follicle. After ovulation, the CL undergoes structural transformation in which both the theca and granulosa cells contribute to progesterone production, resulting in the transformation of the endometrium into a receptive state. If Human Chorionic Gonadotropin (HCG) secretion from an implanting embryo does not occur, CL will undergo luteolysis, leading to a rapid decline in sex steroid levels. The critical role of CL in maintaining early pregnancy was previously clearly demonstrated by an induced pregnancy loss if luteoectomy was performed before the seventh week of gestation [5].

Historically, luteal phase deficiency (LPD) refers to a LP of ≤ 10 days in length [22], however, biochemical definitions have also been proposed. Low P4 levels during the LP is linked to infertility [1, 18] and repeated early pregnancy loss [24]. The P4 production varies significantly among and within individuals, and even in healthy women with regular menstrual cycles, the production varies from cycle to cycle [3, 4, 14, 16]. Schliep et al. studied 259 women over 2 ovulatory cycles, reporting that in 20 out of 463 cycles (4.3%) the duration of the LP was less than 10 days (clinical LPD) and luteal P4 levels were 5 ng/mL or less (biochemical LPD) [23]. Importantly, evidence from true natural cycle frozen embryo transfer suggests that reproductive outcomes are impacted by P4 levels below 10 ng/ml which was seen in 37% of the cycles [9]. Therefore, even if the LPD definitions are not met, suboptimal P4 levels may still be present in regularly menstruating women having a negative impact on reproductive outcomes.

The objective of the present study was to determine whether an intra-individual variability of LP serum P4 levels exists between subsequent cycles and to quantify the extent of variance in secretion pattern and serum P4 levels during three cycles within the same regularly cycling woman. Additionally, we aimed to investigate whether hormone levels and covariates measured during the FP—including Follicle Stimulation Hormone (FSH), Luteinizing Hormone (LH), Estradiol (E2), Anti-Mullerian Hormone (AMH) and Antral Follicle Count (AFC)—could predict the P4 level during the LP, i.e. the function of the CL.

## Material and Methods

A prospective observational study was conducted in a cohort of regularly cycling women with no history of infertility at the Reproductive Medicine Centre at Skane University Hospital Malmö, Lund University, Sweden, between November 2011 and June 2012. Study flow diagram is shown in Figure S1.

### Participants

Potential study subjects were recruited through the use of recruitment posters at the hospital and advertisement in local newspapers, which were directed towards hospital employees and medical or nursing students. Prior to signing the consent form, subjects were required to complete a standardised questionnaire, regarding their health, pregnancies and menstrual cycle length; moreover, oral and written information was given. The inclusion criteria were body mass index (BMI) < 30 kg/m^2^, cycle length between 21–35 days and the exclusion criteria were: use of hormonal medication, a history of infertility, and other gynaecological or chronic medical diseases. Data from the cohort has previously been published [2].

### Blood sampling

The study protocol included blood sampling, starting on the fifth day of the menstrual cycle and continuing every fifth day until the next cycle. The procedure was repeated for a total of three cycles. Blood samplings were exclusively carried out between 08:00 am and 03:00 pm and each blood sample was collected into vacuumed vials containing gel via a heparinised catheter inserted into a forearm vein. Within two hours, the samples were centrifuged at 2,000 g for ten minutes before being stored at a temperature of minus 20 degrees Celsius. Once all samples from a single study subject had been collected, they were transferred and stored at − 80°C until analysis.

### Assays

For FSH, LH, P4 and E2, all samples from one participant were analysed within the same assay run at a Beckman Access Immunoassay System on a UniCelTMDxI800 from Beckman–CoulterInc., Brea, CA, USA. The analyses were performed at Skane University Hospital, Malmö. The lowest detectable level distinguishable from zero with 95% confidence and total coefficient of variances (CV) were; for FSH and LH 0.2 IU/l and CV 9%, for P4 0.25 nmol/l and CV 14%, and for E2 73 pmol/l and CV 13%. All measurements were performed according to the instructions from the manufacturers.

### 3D ultrasound images for antral follicle count

Ultrasound analysis was performed on cycle day 5 using 4D-view™ software, version 9.1 (GE Medical systems, Zipf, Austria) with Sonography-based Automated Volume Calculation (SonoAVC™) software by one observer, and calculations were performed on multi-planar images showing the ovary in the longitudinal, transverse and coronal planes. SonoAVC software was used to calculate the number and size of antral follicles and the average diameter was determined and listed according to the follicular size.

The mean diameter and the number of follicles with a diameter of 2.0—10.0 mm were used for statistical analysis.

### Statistics

Data are presented as medians with interquartile range (IQR), mean with standard division (SD) or numbers with proportions as appropriate, Table 1.

**Table 1:** Descriptive statistics

Variable	Mean (± SD) Median [IQR] Number; proportion
Age, years	27.5 [26; 41]
BMI, kg/m^2^	22.5 [20.8; 23.1]
Weight, kg	64 [59; 69]
AMH cycle day 5, pmol/L	14.1 (± 11.7)
Antral follicle count, n	17.3 (± 12.6)
Cycle length, days	28 [28; 30]
AUC^1^, nmol days/L	269 (± 175)
Number of previous pregnancies, number of women (%)	
0	12; 46%
1	2; 8%
2	6; 23%
≥ 3	6; 23%
Number of previous births, number of women (%)	
0	14; 54%
1	2; 8%
2	8; 31%
≥ 3	2; 8%

^1^Area under the curve. Two cycles were excluded as only one blood sample was analysed during the luteal phase (cycle day 15–30)

A univariate screening of LH, FSH and E2 levels measured on different days, age of the woman, AFC, AMH and weight, was performed to identify factors associated with P4 level ≥ 30 nmol/L on cycle day 20 or 25. In all statistical analyses all cycles were merged, and as participants contributed with data from three cycles and one participant contributed with two cycles, robust standard errors were calculated to account for non-independency of data within the same woman.

The Youden Index was used to define hormone cut-off levels on different blood sampling days with the highest sensitivity and specificity for a P4 ≥ 30 nmol/L on day 20 or 25. The Youden Index is defined as J = max (Sensitivity[c] + Specificity [c] − 1), where c is the cut-off point and the value range from 1 to –1. A Youden Index of 1 represents the perfect test, and a value of 0 or lower than 0 indicates that the test is not fit for use.

#### Estimation of P4 production during the LP

P4 production during the LP was estimated by using area under the curve (AUC) for P4 levels from day 10 until day 25 or day 30, depending on whether participants had bleeding before or after day 30. A definite integral between each P4 measurement after cycle day 10 was calculated and added to define the AUC value.

#### Prediction of P4 levels in the LP

All cycles were included in a logistic regression analysis to examine the relationship between FP hormone levels and LP P4 levels ≥ 30 nmol/L on cycle day 20 or 25, with adjustments made for age, weight, AFC, and AMH (all continuous variables).

The statistical analyses were performed using STATA software, version 16.

### Ethics

The local Ethical Committee at Lund University, Sweden approved the study on 21st of June 2011 (Dnr: 2011/321).

Prior to providing their consent, all participants were given both written and verbal information.

The trial was conducted according to the WMA Declaration of Helsinki and Good Clinical Practice.

## Results

Twenty-six healthy, non-smoking women completed the program. A total of 16 women aged 20–30 years and ten aged 35–50 years were enrolled. Twenty-five women participated with three consecutive cycles and one woman with two cycles, only.

The median age of the 10 women > 35 years was 42 years, interquartile range (IQR) (40;45) and for the 16 women < 30 years the median age was 26 years IQR (25;27). The median cycle length was 28 days, IQR (28; 30), range (25;32).

Basic characteristics are listed in Table 1.

### Serum P4 levels

**Fig. 1:**
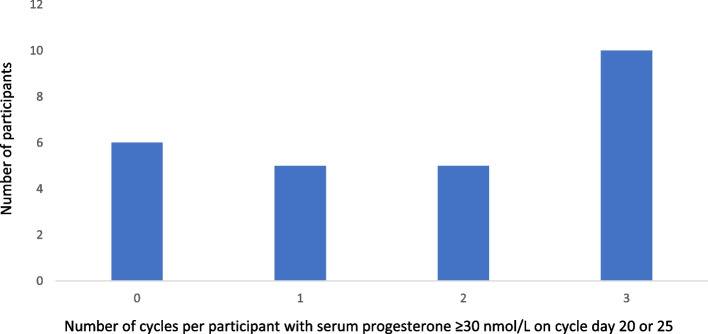
Number of cycles with a progesterone level ≥ 30 nmol/L on cycle day 20 or 25

In total ten women had a P4 level ≥ 30 nmol/L on cycle day 20 or 25 during all three cycles, whereas the remaining 18 women had varying levels of P4 < 30 nmol/L on day 20 and 25. Overall, in 45 out of 77 cycles P4 levels were ≥ 30 nmol/L on day 20 or 25 as illustrated in Fig. 1.

**Fig. 2:**
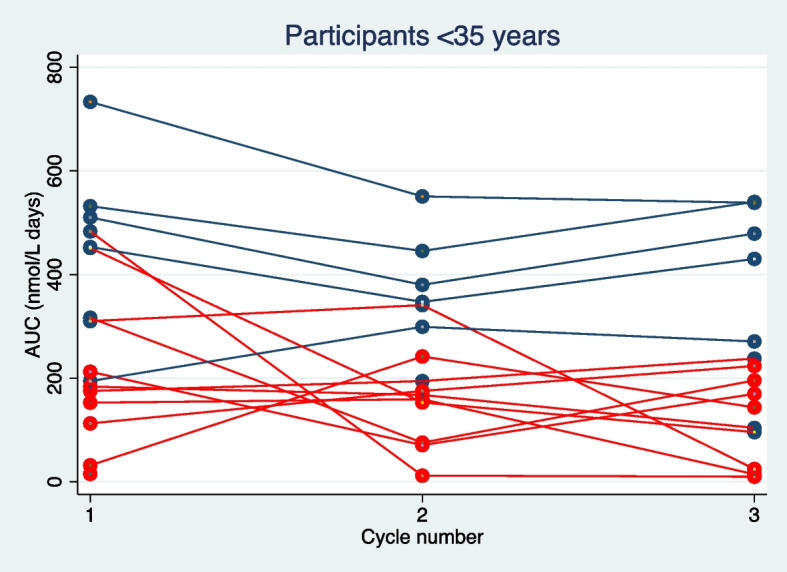
Correlation between AUC (progesterone levels) and a serum progesterone level ≥ 30 nmol/L on cycle day 20 or 25. Participants less than 35 years old. AUC: area under the curve from day 14 to 25 or 30 (progesterone levels). Blue dot represents serum progesterone levels ≥ 30 nmol/L on cycle day 20 or 25. Red dot represents serum progesterone levels < 30 nmol/L on cycle day 20 and 25. Blue line represents a participant with serum progesterone levels ≥ 30 nmol/L on cycle day 20 or 25 in all three cycles. Red line represents a participant with 0, 1 or 2 cycles with serum progesterone levels ≥ 30 nmol/L on cycle day 20 or 25 out of the three cycles

**Fig. 3:**
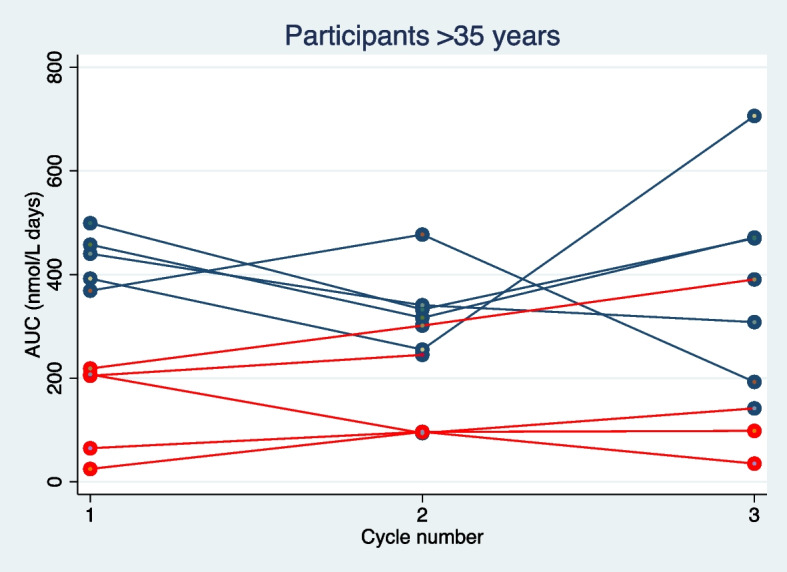
Correlation between AUC (progesterone levels) and a serum progesterone levels ≥ 30 nmol/L cycle day 20 or 25. Participants more than 35 years of age. AUC: area under the curve from day 14 to 25 or 30 (progesterone levels). Blue dot represents serum progesterone levels ≥ 30 nmol/L on cycle day 20 or 25. Red dot represents serum progesterone levels < 30 nmol/L on cycle day 20 and 25. Blue line represents a participant with serum progesterone levels ≥ 30 nmol/L on cycle day 20 or 25 in all three cycles. Red line represents a participant with 0, 1 or 2 cycles with serum progesterone levels ≥ 30 nmol/L on cycle day 20 or 25

The mean of the estimated area under the curve (AUC) was significantly lower in cycles with no P4 levels ≥ 30 nmol/L compared to cycles with a P4 level ≥ 30 nmol/L on day 20 or 25; 118 ± 75 nmol days/L vs. 365 ± 151 nmol days/L (*p* < 0.01), respectively. The correlation between P4 levels ≥ 30 nmol/L on cycle day 20 or 25 and AUC is shown in Figs. 2 and 3.

Figure S2 illustrates the variation in progesterone levels over three cycles for two participants. The data highlights divergent progesterone levels between the cycles.

#### The E2 level on cycle day 5 and 10 during the follicular phase as a predictor of CL function

The E2 cut-off levels on day 5 and 10 with the highest sensitivity and specificity for a P4 ≥ 30 nmol/L on day 20 or 25 defined by the Youden Index were 131 pmol/L and 345 pmol/L, respectively. When comparing cycles with E2 levels ≥ 345 pmol/L on day 10 to cycles with E2 levels < 345 pmol/L significantly more cycles had P4 levels ≥ 30 pmol/L on day 20 or 25, 89% (25/28) versus 45% (20/44), respectively (*p* < 0.01). A significant difference was also found when comparing E2 levels ≥ 131 pmol/L to < 131 pmol/L on cycle day 5, 74% (28/38) versus 45% (17/38), *p* = 0.01. Conclusively, the E2 cut-off level during the FP predicted a P4 level ≥ 30 nmol/L on day 20 or 25, with a sensitivity of 64% and specificity of 69% for cycle day 5 and a sensitivity of 57% and specificity of 89% for cycle day 10.

E2 levels on day 5 and 10 are presented in the univariate logistic analyses for a P4 level ≥ 30nmol/L on days 20 or 25, displayed in both continuous and Youden Index defined categorical data, in Table 2. Multivariate analyses including age, weight, AFC, AMH and E2 levels on day 5 and E2 on day 10, respectively, were performed. In cycles with E2 levels > 131 pmol/l on cycle day 5 the OR for a P4 level ≥ 30nmol/L on day 20 or 25 was 3.8 [1.1; 13.4] (data not shown) and for cycles with an E2 level > 345 pmol/l on cycle day 10, an OR of 10.3 [2.5; 42.4] was found; (Table 3).

**Table 2:** Univariate analysis of parameters related to a serum progesterone level ≥ 30nmol/L on cycle day 25 or 30. All cycles were included

	Odds Ratio [95% CI]	*p*-value
Age^1^, years	1.04 [0.95; 1.15]	0.38
Weight^1^, kg	0.98 [0.92; 1.06]	0.59
AFC^1^, n	0.98 [0.94; 1.03]	0.47
AMH^1^ cycle day 5, pmol/L	0.97 [0.92; 1.03]	0.33
FSH level^1^ cycle day 5, IU/L	0.97 [0.89; 1.06]	0.50
LH level^1^ cycle day 5, IU/L	0.96 [0.85; 1.08]	0.46
LH level^2^ ≥ 11IU/L cycle day 5	0.40 [0.03; 4.70]	0.46
E2 level^1^ cycle day 5	1.01 [1.00; 1.02]	0.03
E2 level^1^ cycle day 10	1.00 [1.00; 1.01]	0.02
E2 level^3^ ≥ 131 pmol/L cycle day 5	3.85 [1.27; 11.68]	0.02
E2 level^4^ ≥ 345 pmol/L cycle day 10	10.96 [3.24; 37.07]	< 0.01

^1^Continuous variables

^2^Categorical variable. Reference group is serum LH levels < 11 IU/L on cycle day 5. The cut-off level calculated by Youden Index

^3^Categorical variable. Reference group is serum E2 levels < 131 pmol/L cycle day 5. The cut-off level calculated by Youden Index

^4^Categorical variable. Reference group is serum E2 levels < 345 pmol/L cycle day 10. The cut-off level calculated by Youden Index

**Table 3:** Multivariate analysis of parameters related to a serum progesterone level ≥ 30nmol/L cycle day 25 or 30. All cycles were included

	Odds Ratio [95% CI]	p-value
Age^1^, years	1.02 [0.90; 1.16]	0.71
Weight^1^, kg	1.04 [0.95; 1.13]	0.37
AFC^1^, n	0.99 [0.90; 1.09]	0.88
AMH^1^ cycle day 10, pmol/L	1.03 [0.95; 1.12]	0.46
E2 level^2^ ≥ 345 pmol/L cycle day 10	10.29 [2.50; 42.41]	< 0.01

^1^Continuous variables

^2^Categorical variable. Reference group is serum E2 levels < 345 pmol/L cycle day 10

### LH levels during the follicular and mid-luteal phases

The LH level on cycle days 5, 10, 15, and 20 had a median of 5.85 IQR (4.7–7.6) IU/L, 6.7 IQR (5.4–8.3) IU/L, 13.7 IQR (8.2–26.5) IU/L and 6.7 IQR (4.1–11) IU/L, respectively. The Youden Index was applied to define LH cut-off values as predictors of a P4 level ≥ 30 nmol/L on day 20 or 25, resulting in cut-off levels of 9.6 IU/L for day 10 and 30.2 IU/L for day 15. However, a significantly higher number of cycles had P4 levels ≥ 30 nmol/L on day 20 or 25 when LH levels were below the cut-off on days 10 and 15 (*p* < 0.03 and *p* < 0.05, respectively).

### FSH levels on cycle day 5

**Fig. 4:**
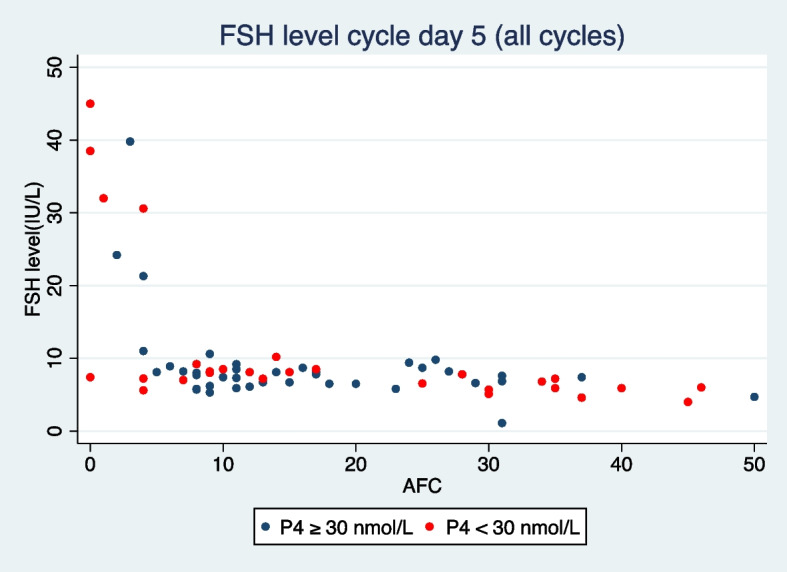
Relation between antral follicle count and FSH levels day 5. Blue dot represents a cycle with serum progesterone levels ≥ 30 nmol/L on cycle day 20 or 25. Red dot represents a cycle with serum progesterone levels < 30 nmol/L on cycle day 20 and 25

The median FSH level on cycle day 5 (all cycles) was 7.6 IU/L with a range of 4 to 32 IU/L IQR (6.4; 8.5) IU/L. The Youden Index identified a cut-off level of 31 IU/L for a P4 level ≥ 30 nmol/L. However, on FSH levels ≥ 31 IU/L were only encountered in five cycles and exclusively in cycles with an AFC ≤ 4. Out of these 5 cycles, only one had a P4 level ≥ 30 nmol/L on day 20 or 25. Figure 4 illustrates the relationship between AFC, FSH levels on cycle day 5, and whether the P4 level in the cycle was ≥ 30 nmol/L on day 20 or 25. Additionally, the FSH levels day 5 did not show a correlation with a P4 level of ≥ 30 nmol/L on day 20 or 25, as indicated in Table 2.

## Discussion

The present study shows highly variable LP P4 levels in healthy regularly cycling women, and furthermore, that the FP E2 level on days 5 and 10 is predictive of a P4 level ≥ 30 nmol/L on days 20 or 25. Importantly, no other covariates, including FSH level cycle day 5, LH levels during the follicular phase, age, weight, AFC and AMH seem to be associated with the LP P4 level.

### The 30 nmol/L luteal phase cut-off for P4

Jordan et al. previously conducted a study in a group of 58 regularly cycling women to explore P4 levels on a daily basis during the LP. The cohort was strictly selected for several parameters, i.e., age, a history of regular menstrual cycles, BMI, and LP length > 12 days. Based on that cohort, luteal phase deficiency (LPD) was defined as an AUC of P4 less than 254.4 nmol days/L during the LP [14]. In another cohort study by Del Pozo et al., LPD was defined as an AUC < 340.26 nmol days/L [21]. Based on these AUC cut-offs and observations, the authors concluded that the most accurate method to predict a low AUC of P4 was one single mid-luteal serum P4 level measurement < 31.8 nmol/L, or the sum of three random serum P4 levels during the LP being less than 95.4 nmol/L.

The AUC in the present study was an estimation based on P4 levels every fifth day, showing significantly lower mean AUC in cycles with no LP P4 levels ≥ 30 nmol/L (118 ± 75 nmol x days/L) compared to a mean AUC of 365 ± 151 nmol days/L in cycles with a P4 level ≥ 30 nmol/L on day 20 or 25.

Interestingly, a study exploring salivary samples consisting of a total of 22 regularly cycling women, demonstrated a significant variation in LP P4 levels between individuals and cycles. The participants collected salivary samples daily over a period of six months, with LP P4 levels calculated as the average of 14 days salivary P4 levels before the onset of menses. The authors focussed on the intra-individual and inter-cycle variation and suggested that changes in calorie intake and physical activity could partially explain this phenomenon [12]. No information on calorie intake or activity was available in the present study, and weight was only measured during the first cycle.

### Is a 30 nmol/L P4 level sufficient to support a pregnancy?

Importantly, the present study did not include contraceptive cycles. The objective of the study was to demonstrate the frequency of cycles with low P4 levels and, by extension, the risk of insufficient P4 levels during the early stages of pregnancy. A sufficient P4 during the luteal phase was evaluated in HRT-FET cycles, with a level of approximately 32 nmol/l being proposed [17]. In relation to the natural cycle, Hull et al. investigated the minimum serum P4 level necessary for a successful live birth to occur in 212 natural conception cycles. The authors concluded that no pregnancy occurred if P4 levels were below 27 nmol/L or above 50 nmol/L. [11]. Thus, LPD is a physiological phenomenon that affects conception during natural cycle. This is particularly important given the increasing use of natural cycle for frozen embryo transfer [26] in which some individuals undergoing true natural frozen embryo transfer (tNC-FET) will need additional exogenous luteal phase support (LPS). Gaggiotti-Marre stressed the point by reporting a significantly higher live birth rate (LBR) if P4 levels exceeded 32 nmol/L, the day prior to blastocyst transfer in tNC-FET cycles, compared to cycles with lower P4 levels (41.1% versus 25.7%, respectively) [9]. However, it appears that LPS involving exogenous P4 may compensate for the P4 deficiency. Thus, Lawrenz et al. reported that if exogenous P4 was added from the blastocyst transfer day in a tNC-FET cycle, P4 levels measured before initiating P4 treatment on the transfer day did not affect the ongoing pregnancy rate [15].

Finally, a meta-analysis (*N* = 489) including three randomised controlled trials showed that exogenous P4 LPS in tNC-FET was superior to no LPS in terms of reproductive outcome with a risk ratio of 1.42 [13].

### E2 levels during follicular phase as a predictor of CL function

E2 is primarily secreted by the ovary due to the aromatization of testosterone in the granulosa cell during the FP. After ovulation, the granulosa cells transform into luteal cells, which secrete P4. Thus, it can be hypothesised that the E2 secretion during the FP mirrors granulosa cell function and if the E2 is high during the FP, the likelihood of an optimal P4 production during the LP is increased. In the present study 89% (25/28) of cycles with an E2 level ≥ 345 pmol/L on day 10 of the FP had a P4 level ≥ 30 nmol/L on days 20 or 25 compared to 45% if E2 was lower than 345 nmol/L (OR 10.0, 95% CI [2.6; 38.4]).

### LH levels as a predictor of CL function

LH plays a crucial role during ovulation, and the LH surge has been described to consist of three phases, an ascending phase, a plateau phase and a declining phase lasting for a total of 48 h [10]. As for the LH peak, Park et al. reported that it could be highly variable in appearance and subsequently different definitions of LH peaks have been suggested [7, 20].

In the present study, lower LH levels on cycle day 10 and 15 correlated with a P4 level ≥ 30 nmol/L on days 20 or 25 compared to higher levels. The paradox may be explained by the fact that most women had their LH peak between the 10th and 15th cycle day and blood sampling in the present study was limited to every fifth day, resulting in small number of LH peaks being detected. Furthermore, during the early LP, P4 is mainly secreted from the large luteal cells (granulosa cells) which are not LH dependent, unlike during the mid- and late LP where the small luteal cells are taking over which are LH and HCG dependent, originating from the theca cells [6, 8, 19].

### FSH or AFC

In the present study, neither FSH levels on cycle day 5 nor AFC were predictive of P4 levels on day 20 or 25. However, due to the small number of women in this cohort (*n* = 6) with AFC < 5 and only 10 cycles (5 patients) in which the FSH level on day 5 was greater than 10 IU/L, we cannot draw any conclusions on whether AFC or FSH are prognostic for P4 levels during the LP.

### Strengths and limitation

The strength of the present study is that 25 women had standardised blood sampling during three consecutive cycles and reduces the risk of changes in lifestyle, including weight and exercise habits which is known to influence steroid levels. Furthermore, all participants were lean, healthy, working women with regular cycles. A limitation is the number of participants and the fact that blood samples were drawn only every fifth day. Ovulation was not detected in the present study, however, as the range of cycle lengths was very narrow (25 to 32 days), the risk of not detecting a mid-luteal P4 rise on either cycle day 20 or 25 is suggested to be low.

With the increasing number of FET cycles being performed in the tNC, we revisited the present data collected during 2011–2012. Importantly, the data question whether all true natural cycles result in optimal LP P4 levels and interestingly, we found that the E2 level on cycle day 10 seems to be a predictor of an optimal FP P4 level. Future research needs to focus on developing diagnostic tools to identify the most optimal natural cycle steroid level.

In conclusion, the present study is the first to suggest a significant correlation between FP E2 levels and a well-functioning CL, secreting a sufficient amount of P4 during the LP. Moreover, our findings again highlight the existence of a significant inter-cycle and intra-individual variation in P4 levels in regularly cycling women. With the increasing use of tNC FET, we underline the need for more research on how to monitor the natural cycle in order to individualise the luteal phase support to achieve the highest reproductive outcome.

### Supplementary Information

Supplementary Material 1.

Supplementary Material 2.

## Data Availability

The datasets used and/or analysed during the current study are available from the corresponding author on reasonable request.
