# Supplementary material for: The natural menstrual cycle revisited – can natural cycle be trusted
Source: J Ovarian Res. 2024 Jul 22;17:153. doi: 10.1186/s13048-024-01469-2 (PMC11265377; doi:10.1186/s13048-024-01469-2)
Supplement: Supplementary file 1 — Supplementary Material 1. [file 13048_2024_1469_MOESM1_ESM.docx]

Figure 1. Study flow diagram.

Assessed for eligibility

n = 27

Enrollment

Allocation

Analysed (n=26)

- Excluded from analysis (n=0)

Excluded due to only one cycle of blood sampling

n = 1

Blood sampling started

n = 27

Analysis

Follow-Up

Lost to follow-up

n=0

Blood sampling completed

n=26

Excluded (n=0)

- Not meeting inclusion criteria (n=0)
- Declined to participate (n=0)
