# Supplementary material for: The natural menstrual cycle revisited – can natural cycle be trusted
Source: J Ovarian Res. 2024 Jul 22;17:153. doi: 10.1186/s13048-024-01469-2 (PMC11265377; doi:10.1186/s13048-024-01469-2)
Supplement: Supplementary file 2 — Supplementary Material 2. [file 13048_2024_1469_MOESM2_ESM.docx]

Figure S2: Progesterone levels for two participants during three cycles.

Serum progesterone level (nmol/L)

Serum progesterone level (nmol/L)
